# GPT-4 generated psychological reports in psychodynamic perspective: a pilot study on quality, risk of hallucination and client satisfaction

**DOI:** 10.3389/fpsyt.2025.1473614

**Published:** 2025-03-19

**Authors:** Namwoo Kim, Jiseon Lee, Sung Hyeon Park, Yoonseo On, Jieun Lee, Musung Keum, Sanghoon Oh, Yoojin Song, Junhee Lee, Geun Hui Won, Joon Sung Shin, Silvia Kyungjin Lho, Yoon Jung Hwang, Tae-Suk Kim

**Affiliations:** ^1^ Department of Clinical Medical Sciences, Seoul National University, Seoul, Republic of Korea; ^2^ Department of Psychiatry, Uijeongbu Eulji Medical Center, Eulji University School of Medicine, Uijeongbu, Republic of Korea; ^3^ Department of Medical Informatics, The Catholic University of Korea College of Medicine, Seoul, Republic of Korea; ^4^ College of Medicine, Ewha Women’s University, Seoul, Republic of Korea; ^5^ Department of Pediatrics, Inje University College of Medicine, Ilsan Paik Hospital, Goyang, Republic of Korea; ^6^ Department of Neuropsychiatry, Hallym University Dongtan Sacred Heart Hospital, Gyeonggi, Republic of Korea; ^7^ Department of Psychiatry, Kangwon National University Hospital, Chuncheon, Republic of Korea; ^8^ Department of Psychiatry, Seoul St. Mary’s Hospital, The Catholic University of Korea, College of Medicine, Seoul, Republic of Korea; ^9^ Department of Psychiatry, Chungnam National University Sejong Hospital, Sejong, Republic of Korea; ^10^ Department of Neuropsychiatry, Seoul National University Hospital, Seoul, Republic of Korea; ^11^ Department of Psychiatry, SMG-SNU Boramae Medical Center, Seoul, Republic of Korea

**Keywords:** artificial intelligence, large language models, gpt, psychodynamic, formulation, hallucination, psychopathology, defence mechanisms

## Abstract

**Background:**

Recently, there have been active proposals on how to utilize large language models (LLMs) in the fields of psychiatry and counseling. It would be interesting to develop programs with LLMs that generate psychodynamic assessments to help individuals gain insights about themselves, and to evaluate the features of such services. However, studies on this subject are rare. This pilot study aims to evaluate quality, risk of hallucination (incorrect AI-generated information), and client satisfaction with psychodynamic psychological reports generated by GPT-4.

**Methods:**

The report comprised five components: psychodynamic formulation, psychopathology, parental influence, defense mechanisms, and client strengths. Participants were recruited from individuals distressed by repetitive interpersonal issues. The study was conducted in three steps: 1) Questions provided to participants, designed to create psychodynamic formulations: 14 questions were generated by GPT for inferring psychodynamic formulations, while 6 fixed questions focused on the participants’ relationship with their parents. A total of 20 questions were provided. Using participants’ responses to these questions, GPT-4 generated the psychological reports. 2) Seven professors of psychiatry from different university hospitals evaluated the quality and risk of hallucinations in the psychological reports by reading the reports only, without meeting the participants. This quality assessment compared the psychological reports generated by GPT-4 with those inferred by the experts. 3) Participants evaluated their satisfaction with the psychological reports. All assessments were conducted using self-report questionnaires based on a Likert scale developed for this study.

**Results:**

A total of 10 participants were recruited, and the average age was 32 years. The median response indicated that quality of all five components of the psychological report was similar to the level inferred by the experts. The risk of hallucination was assessed as ranging from unlikely to minor. According to the median response in the satisfaction evaluation, the participants agreed that the report is clearly understandable, insightful, credible, useful, satisfying, and recommendable.

**Conclusion:**

This study suggests the possibility that artificial intelligence could assist users by providing psychodynamic interpretations.

## 1 Introduction

Large language models (LLMs) like Generative Pre-trained Transformer 4 (GPT-4) have revolutionized technology by generating advanced natural language text, enabling them to perform a variety of complex tasks that previously required human intelligence (1, 2). The emergence of this technology has not only transformed how we interact with computers, making them more intuitive and useful, but has also driven significant advancements in various domains (3).

A variety of ideas for utilizing LLMs in the field of mental health are being proposed. For example, LLMs can help reduce therapists’ workload by collecting and summarizing basic information about clients, providing initial responses, and delivering personalized educational materials that clients may need (4). Furthermore, several studies used LLMs to offer a variety of empathetic and supportive interactions through conversations with the model (5–8).

In particular, providing clients with psychologically profound insights would be one of the key objectives of using LLMs. Among the diverse methods of providing insights, psychodynamic psychotherapy, which is characterized by deeply exploring an individual’s unconscious motives and internal conflicts, going beyond simple behavioral modifications to address fundamental psychological conflicts, may be particularly suitable (9). However, the prevailing opinion among researchers is that LLMs have not yet reached the level needed to replace counselors (10). For example, LLMs do not adequately perform tasks such as determining contextual relevance, understanding temporal dynamics, achieving depth in emotional comprehension, and maintaining consistency across conversations (11–13). Considering these limitations of current LLMs, generating a psychodynamic formulation could be an alternative. A psychodynamic formulation is a way to understand a person’s emotional and behavioral patterns by exploring their past experiences, relationships, and unconscious processes (14). It examines how early experiences, such as childhood relationships or family dynamics, influence their thinking, feelings, and interactions. It also considers internal struggles, like the tension between desires and obligations, and coping mechanisms. This approach helps clarify why someone feels stuck or distressed and guides therapy to promote self-awareness, emotional growth, and healthier relationships. Using data from a psychodynamic perspective regarding a client, it may be possible to generate a psychodynamic formulation that provides psychological insights to the client using LLMs.

However, studies that use LLMs to generate psychodynamic formulations and explore their quality or the presence of hallucinations (incorrect AI-generated information) are rare (15). Previously, there was a study examining the features of psychodynamic formulations generated by ChatGPT using data from psychoanalytic literature (15). However, this study did not directly recruit participants to obtain data from them and also had the limitation of using the GPT-3.5 model, which is significantly less capable than the current, more advanced GPT-4 model.

This pilot study aims to generate psychodynamic formulations using GPT-4 based on texts written by participants, and to evaluate their quality, risk of hallucination, and participant satisfaction. Through this study, we explore the possibility that artificial intelligence (AI), with minimal human intervention, can provide meaningful psychodynamic interpretations that offer individuals new insights into their psychological issues.

## 2 Methods

### 2.1 Study participants

The inclusion criteria required participants (‘the clients’) to be experiencing psychological difficulties due to recurrent interpersonal issues and to be capable of using computers for document work and sending/receiving emails via the internet. The exclusion criteria included individuals with severe symptoms such as depressive mood, anxiety, hallucinations, delusions, or suicidal impulses. The methods for recruiting research participants involved posting advertisements in the form of posters at Seoul St. Mary’s Hospital and the Catholic University, where the lead authors work. Individuals interested in participating in the study contacted the authors after seeing these posters. The anticipated audience for the posters included patients, their guardians, hospital staff, and students who were not directly or indirectly associated with the authors. The evaluation of inclusion and exclusion criteria was conducted through a brief interview between individuals wishing to participate in the study and the author, who is a psychiatrist, without the use of assessment tools. The authors instructed the clients not to include sensitive personal information in their writings, as it would be input into GPT-4, and also informed them of the potential use of their data by OpenAI (16). Only those who consented participated in the study.

This study involved the expertise of seven professors of psychiatry from various university hospitals to evaluate quality of the psychological reports and potential risk of hallucinations. They all had at least four years of experience as therapists in psychoanalytic psychotherapy. To minimize bias in assessing the reports, none of the experts were affiliated with St. Mary’s Hospital, where the lead authors of the study are employed. In addition, to allow for diverse opinions, each expert was recruited from different university hospitals. The authors did not inform the experts that the psychological reports were generated by AI until after the evaluation was complete. Personal information of the clients such as the name, age, and sex was not provided to the experts. The clients agreed to have their writings and the psychological reports read and evaluated by the experts. The study commenced in December 2023 and concluded in June 2024. It was approved by the Institutional Review Board of Seoul St. Mary’s Hospital (IRB number: KC24QISI0057), and informed consent was obtained from all participants.

### 2.2 Study design and process

This study focused on evaluating a psychodynamic formulation generated by GPT-4. In addition to the psychodynamic formulation, the authors further generated other components that may be useful in expanding clients’ psychological insights. Under the psychodynamic perspective, the authors generated psychopathology, parental influence, defense mechanisms, and client strengths with GPT-4. The authors will refer to the combination of these five components, including the psychodynamic formulation, as a psychological report.

**Figure 1:**
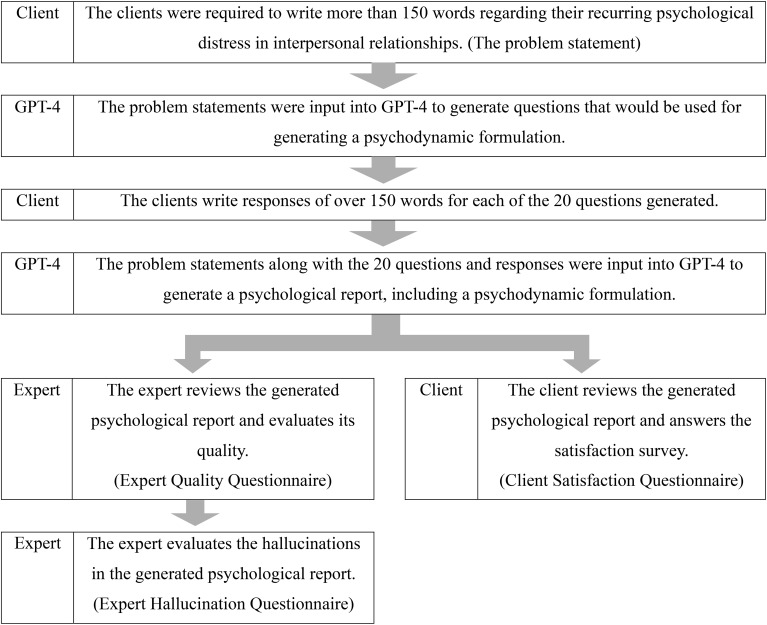
Study flow.

This study was organized into three steps as follows (**Figure 1**); 1) GPT-4 generates a psychological report based on the text provided by the clients; 2) The experts assess quality of the psychological report and the risk of hallucinations; 3) The clients evaluate their satisfaction with the psychological report.

#### 2.2.1 Generating the psychological reports with GPT-4

To generate a psychodynamic formulation, GPT-4 requires appropriate data. This study obtained such data through a process of ‘questions and answers’. Specifically, the authors prepared questions suitable for inferring the psychodynamic formulation, presented them to the clients, and then input the clients’ responses into GPT-4 to generate the psychodynamic formulation. Some of the questions were fixed questions about the participants’ relationship with their parents, while others were generated by GPT for inferring psychodynamic formulations.

The detailed process is as follows. First, the clients described the recurring interpersonal suffering they had recently been experiencing in a statement exceeding 150 words (referred to as the ‘problem statement’). This was input into GPT-4 to generate 14 questions that would be used to infer the psychodynamic formulation. Additionally, the authors created six more questions, making a total of 20 questions. These six questions, which inquire about the client’s relationship with their father and mother, were considered essential for inferring the psychodynamic formulation (refer to **Supplementary Material p2** for the six questions). While these six questions were presented identically to all clients, the 14 questions generated by GPT-4 vary for each client. If there were questions among the 20 that were similar in meaning, the clients still answered all of them. The clients were required to answer each of the 20 questions with more than 150 words. An instruction was provided to guide the clients to answer as freely and frankly as possible (refer to **Supplementary Material p3**).

Next, using the client’s problem statement and the 20 questions and their answers, GPT-4 generated the psychological report including psychodynamic formulation. Among the five components that make up the psychological report, the parental influence was generated from a psychodynamic perspective to explain the positive and negative effects that interactions with parents had on the client. The defense mechanisms described the types of defense mechanisms commonly used by the client, along with their adaptive and maladaptive aspects All prompts entered into GPT-4 for generating the psychological report were described in the **Supplementary Material p4**. For instance, the prompt used to generate the psychodynamic formulation, which is central to this study, consisted of the following three steps: (1) Be sure to read the entire document and respond. Write a psychodynamic formulation for the client. (2) Integrate into one paragraph without losing any content. (3) Write more insightfully and at greater length. Additionally, the authors have included a psychological report in the **Supplementary Material**, written by one of the team members who is not the client, to provide readers with a practical example. (**Supplementary Material pp17-70**). The research process was conducted through email exchanges between the clients and the research assistant. For instance, the research assistant would send 20 questions to the client, who would then provide responses. The assistant would input these responses into GPT to generate a psychological report.

#### 2.2.2 Evaluation of quality and hallucinations in the psychological reports by the experts

The authors provided the experts with the clients’ problem statement, 20 questions and answers, and their psychological reports. The experts responded to a questionnaire regarding quality of each psychological report (Expert Quality Questionnaire). In the survey, the criterion for the quality was based on how validly, specifically, and comprehensively the report describes the psychological, emotional, and behavioral characteristics of the client. After the experts submitted their responses to the authors, they were informed by the authors that both the 20 questions and the psychological reports were generated by GPT-4. The experts did not write the psychological reports themselves. Instead, they read AI-generated reports and evaluated, based on the given criteria. Subsequently, the experts answered a survey related to hallucinations in the psychological reports (Expert Hallucination Questionnaire). In this study, ‘hallucinations’ were defined as distinct errors or clearly false statements in the psychological reports. It is important to note that the experts did not meet the clients directly. Instead, they reviewed the psychological reports generated by GPT and assessed their quality and risk of hallucination. Surveys were also conducted via email.

#### 2.2.3 Evaluation of client satisfaction with the psychological report

The authors provided the clients with their psychological reports, and within one week, the clients responded to a satisfaction survey regarding the reports (Client Satisfaction Questionnaire).

### 2.3 Measures

Three questionnaires were used in the study, all of which were developed by the authors for this study (**Table 1** for summary, and **Supplementary Material pp8-16** for full version).

**Table 1:** Summary of the three survey questionnaires.

Expert Quality Questionnaire (summary)	
1	Evaluate the quality of the psychodynamic formulation described in the psychological report by comparing it to the psychodynamic formulation you have inferred.^1), 2)^
2	Evaluate the quality of the psychopathology described in the psychological report by comparing it to the psychopathology you have inferred.^1), 2)^
3	Evaluate the quality of the parental influence described in the psychological report by comparing it to the parental influence you have inferred.^1), 2)^
4	Evaluate the quality of the defense mechanisms described in the psychological report by comparing them to the defense mechanisms you have inferred.^1), 2)^
5	Evaluate the quality of the client strengths described in the psychological report by comparing them to the client strengths you have inferred.^1), 2)^
6	The psychodynamic formulation is appropriately written.^1), 3)^
7	The psychopathology is appropriately written.^1), 3)^
8	The parental influence is appropriately written.^1), 3)^
9	The defense mechanisms are appropriately written.^1), 3)^
10	The client strengths are appropriately written.^1), 3)^
11	The psychodynamic formulation exhibits elaboration and complexity.^3), 4)^
12	The psychodynamic formulation describes the client as a unique individual.^3), 5)^
13	The psychodynamic formulation is coherent.^3), 6)^
14	The 20 questions have been appropriately selected to infer the client's psychodynamic formulation.^1), 3)^

Expert Hallucination Questionnaire (summary)	
1	Number of distinct errors or clearly false statements per psychological report.^7)^
2	Degree of harm that the distinct errors or clearly false statements in the psychological report could cause to the client.^8)^

Client Satisfaction Questionnaire (summary)	
1	Clearly understood the contents of the psychological report (Understandable).^3)^
2	Gained or expanded my understanding of myself (Insightful).^3)^
3	Trusted the contents of the psychological report (Credible).^3)^
4	Found the psychological report to be useful (Useful ).^3)^
5	Generally satisfied with the psychological report (Satisfying).^3)^
6	Willing to recommend this AI program to a friend who needs similar help (Recommendable).^3)^
7	Preference between psychological report services provided by artificial intelligence and by a human.^9)^

Refer to the **Supplementary Material** for the full version.

^1)^The criteria for the ‘quality’ and the ‘appropriately’ are how validly, specifically, and comprehensively the text describes the psychological, emotional, and behavioral characteristics of the client.

^2)^Likert scale: (1) Much lower than the level I had inferred. (2) Slightly lower than the level I had inferred. (3) Similar to the level I had inferred. (4) Slightly better than the level I had inferred. (5) Much better than the level I had inferred.

^3)^Likert scale: (1) Strongly disagree. (2) Disagree. (3) Agree. (4) Strongly agree.

^4)^The term 'elaboration and complexity' refers to the degree to which various aspects of the client are integrated into a comprehensive and detailed explanation.

^5)^This refers to whether the psychodynamic formulation reflects the unique characteristics of the client, such as their distinct personal experiences and conflicts, rather than being filled with general and theoretical content.

^6)^The coherence refers to how internally consistent the formulation was in explaining an individual’s problems.

^7)^If less than 1, it was described using decimals. For example: 0.1, 0.3, 0.5, etc.

^8)^Likert scale: (1) Unlikely to cause harm. (2) Potentially cause minor harm. (3) Potentially cause moderate harm. (4) Potentially cause serious harm.

^9)^Options: (1) Prefer artificial intelligence. (2) Prefer a human. (3) Both are equally prefered.

First, the Expert Quality Questionnaire is a 14-item survey designed for the experts to evaluate quality of the psychological reports. Options for answering the questions comparing the quality of components such as psychodynamic formulations inferred by the experts and those generated by GPT-4 are as follows: (1) Much lower than the level I had inferred; (2) Slightly lower than the level I had inferred; (3) Similar to the level I had inferred; (4) Slightly better than the level I had inferred; (5) Much better than the level I had inferred. This study referenced previous study on the quality of psychotherapy case formulations (17). Specifically, elements such as ‘elaboration and complexity’, ‘coherence’, and ‘client as a unique individual’ used in that study were included in this survey.

Second, the Expert Hallucination Questionnaire is a two-item survey designed to assess the number of hallucinations identified per psychological report and evaluate the potential harm of the hallucinations.

Third, the Client Satisfaction Questionnaire is a seven-item survey designed to assess the clients’ satisfaction with the psychological reports. The first six questions investigate whether the report is clearly understandable, insightful, credible, useful, satisfying, and recommendable. The response options for these questions are as follows: (1) Strongly disagree; (2) Disagree; (3) Agree; (4) Strongly agree. The final question explores clients’ preferences for receiving these reports, whether through AI or face-to-face interactions.

### 2.4 Outcomes

The study descriptively analyzed the sociodemographic characteristics of the clients and the experts. It detailed the results of the three surveys, presenting data on frequency, median, average, or range for each item. The median response to each question was used as the primary outcome. All clients and experts had a response rate of 100%.

## 3 Results

In **Table 2**, the sociodemographic characteristics of the clients and the experts are described. There was a total of 10 clients, with an average age of 32 years; 3 were male and 7 were female. The average number of years of education was 16.4. There were 7 experts in total, with an average age of 36.6 years; 4 were male and 3 were female. The average number of years of experience as psychiatrists was 9.3 years.

**Table 2:** Sociodemographic characteristics of the participants.

The Clients	
n	10
Age (years); mean (range)	32 (29-38)
Sex (male/female)	3/7
Years of education(years); mean (range)	16.4 (12-18)

The Experts	
n	7
Age(years); mean (range)	36.6 (35-41)
Sex (male/female)	4/3
Years of experience as psychiatrist (years); mean (range)	9.3 (8-12)

**Figure 2:**
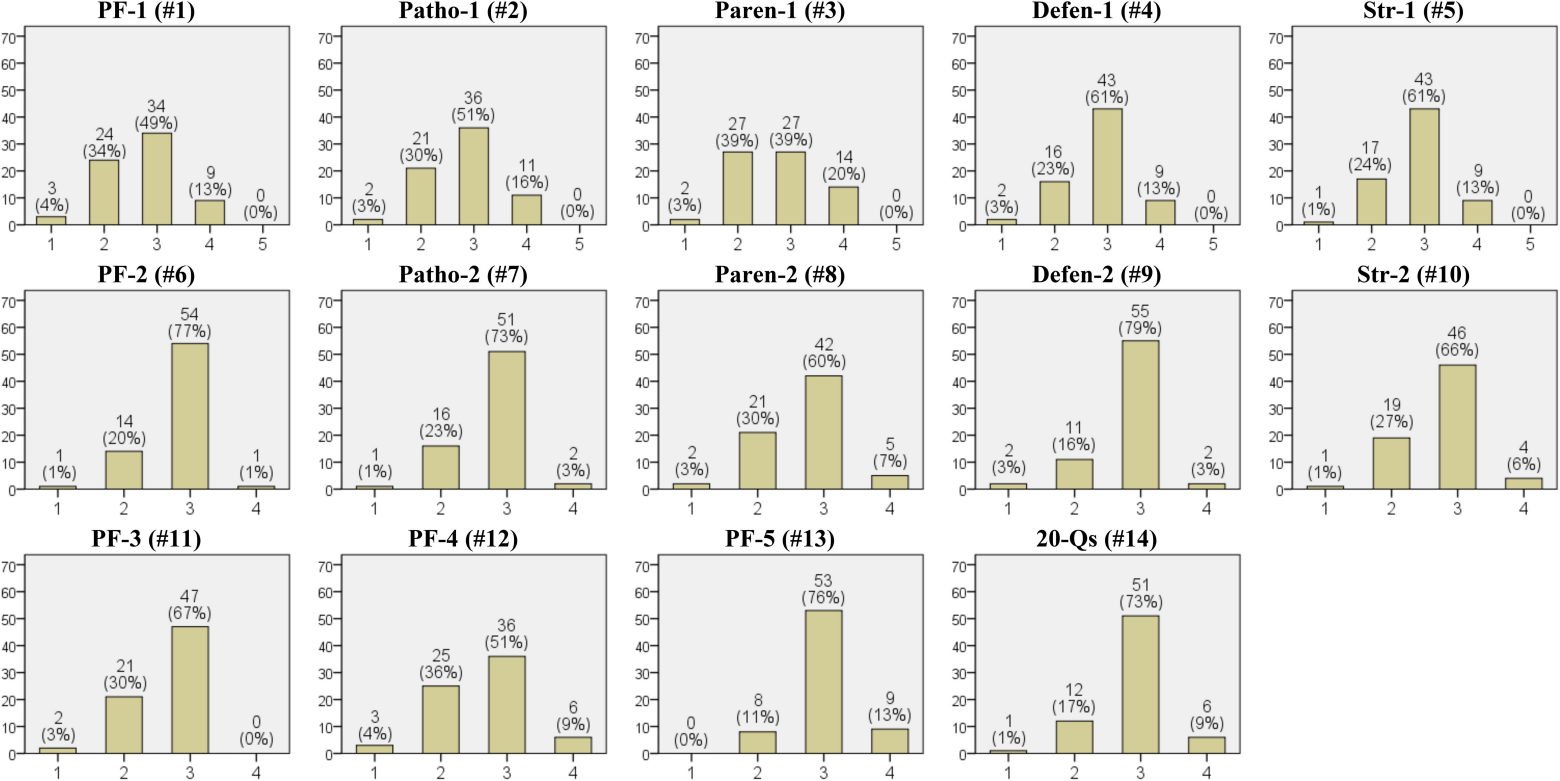
Results of the expert quality questionnaire. The numbers in parentheses in the titles of each graph correspond to the question numbers from the ‘Expert Quality Questionnaire’. The x-axis represents the options for each question, and the y-axis represents the frequency (7 experts each evaluated 10 psychological reports, resulting in a total of 70 evaluated psychological reports). PF, Psychodynamic Formulation; Patho, Psychopathology; Paren, Parental Influence; Defen, Defence Mechanisms; Str, Client Strengths; 20-Qs, the 20 questions generated. Options for questions #1-5 are on a Likert scale: (1) Much lower than the level I had inferred. (2) Slightly lower than the level I had inferred. (3) Similar to the level I had inferred. (4) Slightly better than the level I had inferred. (5) Much better than the level I had inferred. Options for questions #6-14 are on a Likert scale: (1) Strongly disagree. (2) Disagree. (3) Agree. (4) Strongly agree.

In **Figure 2**, the results of the expert evaluations regarding quality of the generated psychological report are presented (Expert Quality Questionnaire). Questions 1 through 5 evaluate the quality of GPT-4’s inferences compared to those inferred by the experts regarding five components of the psychological report: psychodynamic formulation, psychopathology, parental influence, defense mechanisms, and client strengths. In all five components, the median response was ‘(3) Similar to the level I had inferred’, which was also the most frequent response. For parental influences, both ‘(3) Similar’ and ‘(2) Slightly lower’ were reported with equal frequency. Notably, ‘(5) Much better’ was not reported for any of the five components. Questions 6 through 10 assess whether the generated five components were appropriately addressed, with both the median and most frequent response showing ‘Agree’. Questions 11 through 13 evaluate whether the psychodynamic formulation incorporates elaboration and complexity, coherence, and uniqueness, showing ‘Agree’ as both the median and the most frequent response. The final question, number 14, asked whether the 20 questions chosen for the psychodynamic formulation inference were appropriate. The median and the most frequent response were both ‘Agree’.

In **Table 3**, the results of the experts’ evaluations of the hallucinations in the generated psychological reports are described (Expert Hallucination Questionnaire). The median, mean, and range of the number of hallucinations per psychological report were 0.3, 1.3, and 0.1-5, respectively. Regarding the potential harm caused by hallucinations, four out of seven experts (58%) responded with ‘(1) Unlikely to cause harm’, three experts (42%) indicated ‘(2) Potentially cause minor harm’, and no experts reported a likelihood of moderate or severe harm.

**Table 3:** Results of the expert hallucination questionnaire.

#1. Number of hallucinations per psychological report ^1)^	
Median, Mean (Range)	0.3, 1.3 (0.1-5)

#2. Potential harm caused by hallucinations ^1), 2)^	
(1) Unlikely to cause harm.	4 (58%)
(2) Potentially cause minor harm.	3 (42%)
(3) Potentially cause moderate harm.	0 (0%)
(4) Potentially cause serious harm.	0 (0%)

Responses from seven experts to the questions of the Expert Hallucination Questionnaire.

^1)^Hallucination refers to distinct errors or clearly false statements.

^2)^Frequency of responses among the seven experts.

**Figure 3:**
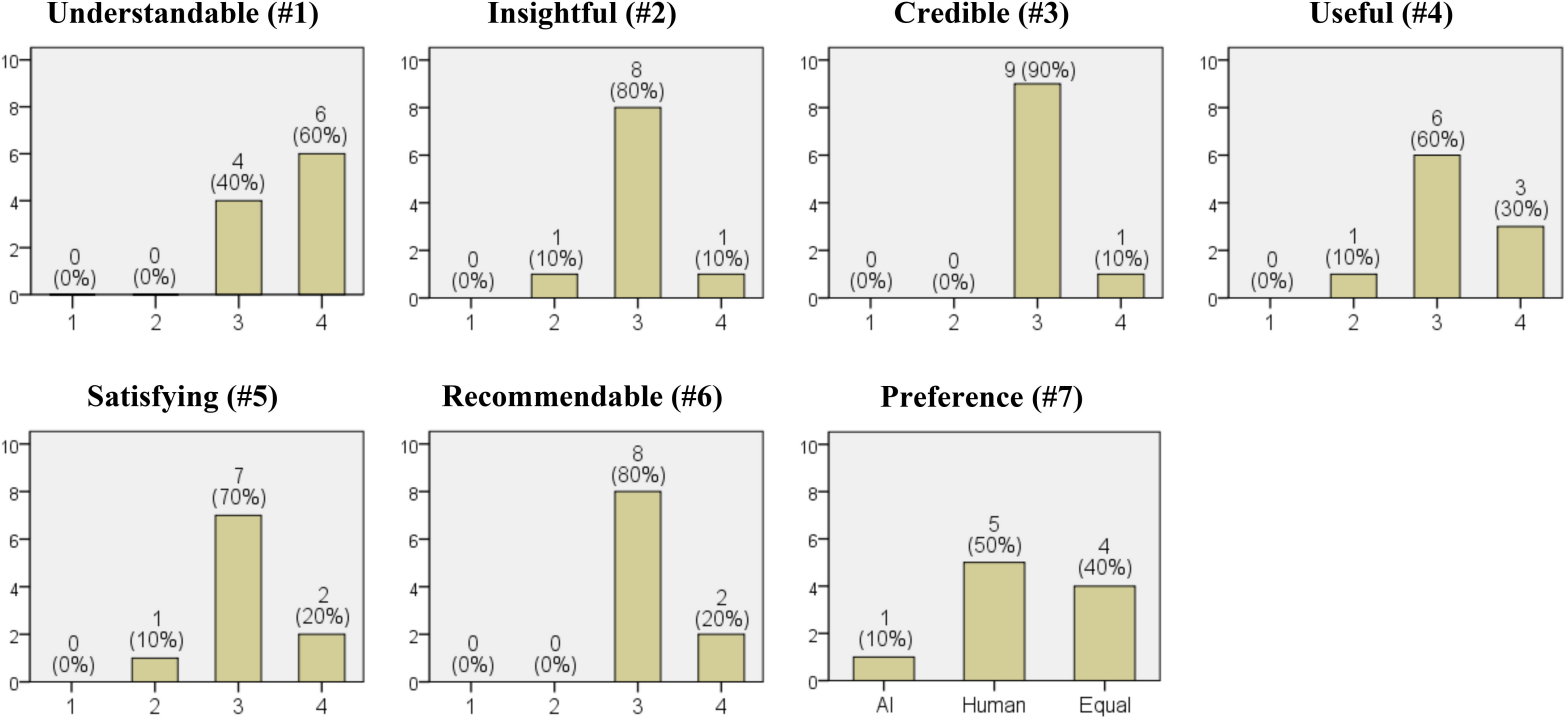
Results of the client satisfaction questionnaire. The numbers in parentheses in the titles of each graph correspond to the question numbers from the 'Client Satisfaction Questionnaire'. The x-axis represents the options for each question, and the y-axis represents the frequency (from a total of 10 clients). Options for questions #1-6 are on a Likert scale: (1) Strongly disagree. (2) Disagree. (3) Agree. (4) Strongly agree. Options for question #7: (1) Prefer AI. (2) Prefer a human. (3) Both are equally prefered.

In **Figure 3**, the results regarding the clients’ satisfaction with the generated psychological reports are presented (Client Satisfaction Questionnaire). For questions 1 through 6, the clients responded on whether the reports were clearly understandable, insightful, credible, useful, satisfying, and recommendable. The median and the most frequent response were either ‘Agree’ or ‘Strongly agree’. Regarding the preference for the service providing the psychological reports, one out of ten respondents preferred it to be delivered by AI (10%), five preferred human providers (50%), and four had no preference between the two (40%).

## 4 Discussion

This study generated psychological reports that primarily focus on psychodynamic formulation, using LLM (GPT-4) for the clients experiencing psychological distress due to recurring interpersonal issues. Furthermore, it presents the evaluations of these reports’ quality and the risks of hallucinations by university hospital professors of psychiatry, along with the results of client satisfaction survey. The findings of this study indicated that the quality of all five components of the generated psychological reports (psychodynamic formulation, psychopathology, parental influence, defense mechanisms, and client strengths) might be comparable to those inferred by the experts. The potential harm caused by hallucinations was found to be unlikely or minor. The clients generally expressed satisfaction with the psychological reports. Readers can directly evaluate the quality and satisfaction by reading an example of the generated psychological report (**Supplementary Material pp17-60**).

When planning this study, the authors considered what data to use for generating psychodynamic formulations with GPT-4. One approach involved using actual counseling conversations between clients and counselors. These conversations would be recorded, transcribed into text, and then input into GPT-4 to generate psychodynamic formulations. However, since counseling sessions often include conversation on routine or transient issues besides the information needed for generating psychodynamic formulations, the authors anticipated that many hours of dialogue would be required. With current advances in LLMs, it’s now possible to input substantial amounts of tokens at once (18). Yet, a more crucial issue is the need to preprocess the data necessary for generating psychodynamic formulations, as the quality of LLM inferences is highly dependent on the quality of the data (19). Therefore, future research should focus on methods that enable AI to automatically perform data preprocessing to generate excellent psychodynamic formulations. Furthermore, as actual counseling conversations are used, measures to protect the client’s personal information will be essential.

Another approach that was considered is using GPT-4 to conduct real-time counseling conversations with clients. This would require preparation such as prompt engineering that enable GPT-4 to engage in appropriate conversations for this purpose. We attempted this method, but GPT-4 faced significant limitations. For example, the level of GPT-4’s ability to deeply explore conflicts within a single conversational topic, infer the client’s unconscious desires in context, or recall relevant content from the client’s past statements related to the current topic, was unsatisfactory. Given these challenges, the authors considered a more structured approach: provide clients with questions that evaluate them psychodynamically and use their responses to generate psychodynamic formulations.

Specifically, in this study, the questions used for generating psychodynamic formulations consisted of fixed questions created by the authors and additional questions generated by GPT-4, and the experts evaluated whether these questions were appropriate for inferring psychodynamic formulations. According to the results, 81% (57 out of 70) of the experts responded with ‘Agree’ or ‘Strongly agree’ to the appropriateness of the questions (**Figure 2**. #14). However, this study did not investigate whether the experts’ evaluation of the appropriateness of the questions pertained to those generated by GPT or the fixed questions created by the authors. Therefore, caution is necessary when interpreting this aspect. Meanwhile, the quality of the generated psychodynamic formulation is likely influenced significantly by the quality of the questions. In this study, the required number of questions was arbitrarily chosen, with each requiring more than 150 words in the response, which was a significant volume. The authors noted that it took at least four hours to complete this task, requiring high concentration. Additionally, upon reviewing the results of the generated psychological report, it appears that GPT-4 utilized only portions of the responses to the 20 questions for its inferences. Thus, future research is necessary to identify the most suitable prompts for generating the questions, the appropriate number of questions, and the word count required for each answer.

In this study, the experts were tasked with devising their own psychodynamic formulations based on the provided questions and answers, then comparing these with those generated by GPT-4. If the experts had directly met and interacted with the clients, developed the questions for psychodynamic formulation inference themselves, and derived the psychodynamic formulations based on the clients’ responses, the quality of these formulations might have significantly surpassed those generated by GPT-4 based on 20 questions. Therefore, it is essential to exercise great caution in interpreting the results of this study.

We think that multiple capabilities are necessary for AI to produce high-quality psychodynamic formulations. These include a robust knowledge base in psychology (especially psychoanalysis and psychodynamic theory), contextual understanding and integrative reasoning capabilities, proficient summarization of large-scale text and extraction of core information, and sophisticated linguistic expression and narrative construction skills. Among these, we consider reasoning to be particularly critical. This is because high-quality psychodynamic formulations require the AI to not only list psychoanalytic terminologies or factually summarize user data but also integrate psychodynamic concepts and extensive client information into a coherent narrative while maintaining logical reasoning throughout. Recent research comparing OpenAI’s o1-preview model to humans in higher-order cognitive domains—including critical thinking, systematic thinking, logical reasoning, and scientific reasoning—found that the model outperformed humans in certain areas (20). Future studies should investigate the quality of psychodynamic formulations generated by more advanced models.

Research on the use of LLMs across various fields is advancing rapidly (21). However, the accuracy and reliability of the information they provide remain significant challenges for practical application. In fields like psychological counseling and psychiatry, however, LLMs can be uniquely valuable. We believe that their ability to present diverse perspectives and possibilities aligns well with the goals of counseling, which seek to explore clients’ experiences and emotions comprehensively, rather than merely seeking correct answers. For instance, a person’s identity is not static and singular; it is dynamic and shaped through interactions with various factors (22). An individual can also experience a mix of emotions or judgments at the same time, and both positive and negative interpretations of social interactions can be valid and convincing. Given these characteristics, pursuing only a precise interpretation of a situation could actually be harmful to the client. Therefore, the generative psychological reports of GPT-4 can expand interpretations about clients and offer counselors or psychiatrists creative or curious perspectives worth considering. Perhaps it is due to these features that the experts in this study rated the risk of hallucinations in the psychological reports as ‘unlikely’ or ‘minor’.

Nevertheless, upon examining the generated psychological reports by the authors, it was observed that there were interpretations that might be difficult for the clients to easily accept. For example, in the case of a client raised by a strict and controlling mother, GPT-4 suggested that overcoming the pain from interactions with her mother could have developed the client’s patience and enhanced emotional regulation skills, which could be seen as a positive influence from the mother. While this interpretation is worth considering, presenting such a perspective abruptly to a client filled with traumatic memories of their mother could provoke severe discomfort and anger. In this context, it is important to consider the necessity of providing psychological reports generated using LLMs under the supervision of psychotherapists or psychiatrists. Alternatively, these generated reports could be used as an initial assessment tool to reference key conflicts, core psychopathologies, and defense mechanisms of the client. Professionals may need to appropriately modify the interpretations raised by LLMs in a way that aids the psychological stability and therapeutic process of the client, and provide supplementary explanations to the client when necessary.

The results of the client satisfaction survey indicated that 4 out of 10 clients preferred both AI and human interaction equally (**Figure 3**. #7). This suggests that the services provided by AI may hold significant potential worth considering. They may place high value on the benefits of remote services, which are not constrained by time or location. Although this study did not explore the reasons behind this preference, the results highlight the potential for developing psychological report services through non-face-to-face means using LLMs. Furthermore, beyond generating psychodynamic formulations, it is possible to explore various psychological features using LLMs. For example, one could infer clients’ personalities, temperaments, or attachment styles, or attempt to understand clients from a perspective other than psychodynamic theory, such as the humanistic theory. The quality evaluations were also very encouraging. Although there were no instances where the generated psychological reports were evaluated as ‘much better’ than the experts’ inferences, ‘slightly better’ accounted for 13%-20% of the cases (**Figure 2**). Considering that the experts are professors of psychiatry currently working in university hospitals, this is an interesting result.

This study has several strengths. It is the first study to generate a psychological report centered on psychodynamic formulation using GPT-4, based on texts written directly by participants about their psychological distress. Additionally, this study evaluated the quality and risk of hallucinations of the reports through expert reviews, and investigated client satisfaction to better capture the features of such services. Further, this study demonstrated the potential for this service to be provided automatically with minimal human intervention by showing that GPT-4 can generate not only psychodynamic formulations but also the necessary questions for making such inferences.

However, this study has the following limitations. First, the number of the clients and the experts involved in this study is small, making it difficult to fully trust the survey results. Despite this, the results of this study suggest the potential for use as foundational data in planning future larger-scale studies. Second, the three questionnaires used in the study are not validated measures, which means the results lack sufficient validity and reliability. In this study, we applied an approach that compares the skills of human experts with those of GPT to evaluate the quality of reports. The criteria for “quality” were presented to the evaluators as how validly, specifically, and comprehensively the text describes the psychological, emotional, and behavioral characteristics of the client. However, no clear definitions or standards for validity, specificity, and comprehensiveness were provided. This represents a significant limitation of the study, which substantially undermines the validity and reliability of its findings. Future studies could address these issues using the following methods. First, other experts could conduct blind evaluations of psychodynamic formulations written by both human experts and AI. Second, to clarify the evaluation criteria, evaluators could be educated on the definitions, standards, evaluation guidelines, and examples for validity, specificity, and comprehensiveness, ensuring a consistent basis for assessment. Third, the Delphi method could be applied (23). This forecasting technique gathers and consolidates expert opinions through iterative surveys and feedback, often used for addressing complex or uncertain issues. Through this consensus-building process, new evaluation tools could be developed, and discussions among expert groups about the quality of psychodynamic formulations could lead to gradual convergence. This process would involve experts sharing their subjective views on concepts like validity and coherence, engaging in open questions and revisions, and arriving at an agreed-upon set of tools and evaluation results. Lastly, involving a diverse group of experts would be crucial. A significant limitation of this study was that the expert group consisted solely of psychiatrists. Although these psychiatrists were university professors with over four years of psychoanalytic education, their perspectives might differ from full-time counselors or psychotherapists. Additionally, it would be beneficial to consider input from experts specializing in other fields, such as cognitive-behavioral therapy. This raises the question of whether the current study’s expert group truly represents “experts.” (17) Stricter criteria for defining expertise in psychodynamic formulation need to be established. For example, experts could be required to have authored books or papers on psychotherapy or led psychotherapy conferences or workshops. Future research should undertake these processes to enhance the validity and reliability of the tools and evaluation outcomes. Third, the clients of the study were limited to individuals currently experiencing psychological distress due to repeated interpersonal problems. This limitation was necessary because the purpose and components of the psychological reports must vary depending on the main issues of the client. For example, for clients suffering from trauma-related distress due to disasters, LLMs services that manage trauma symptoms might be more appropriate than services providing psychodynamic formulations. In the future, more advanced LLMs are expected to be developed to offer flexible and extensive services that provide personalized assistance for a range of psychological problems. Forth, if the clients already possessed a high level of psychodynamic insight about themselves and, as a result, provided responses with high insight to the 20 questions, this could have potentially improved the quality of the psychodynamic formulations generated by the AI. In other words, it is necessary to consider and control the clients’ psychodynamic insight as a confounding factor; however, this was not implemented in the present study. Lastly, the psychological reports generated by GPT-4 can vary with each generation due to the inherent nature of LLMs. Additionally, the performance of the GPT-4 model may change at any time based on decisions by OpenAI. Consequently, there are challenges in reproducing research results, and consistently guaranteeing the quality of the psychological reports is difficult. To address these issues, future study should consider using open source LLMs, or developing AI models that evaluate the quality of generated psychological reports, ensuring that only reports meeting a certain quality threshold are provided.

This study explores whether the innovative technology of LLMs can assist users in understanding themselves psychodynamically. Despite limitations such as the small number of participants and the use of measurement tools that have not yet been validated, considering the overall satisfactory experience of the clients with the psychological reports, the quality comparable to that of the experts, and the low risk of hallucinations, the potential for the development of LLM-based services in the field of counseling or psychiatry appears very high. Furthermore, given the characteristics of being unconstrained by time and space, it is expected to contribute significantly to the enhancement of mental health for many more individuals.

## Data Availability

The datasets presented in this article are not readily available because The dataset used in this study, which consists of texts written by participants from a psychodynamic perspective about themselves, cannot be shared due to privacy concerns. Requests to access the datasets should be directed to Namwoo Kim, rimbaud13@gmail.com.
