# Supplementary material for: GPT-4 generated psychological reports in psychodynamic perspective: a pilot study on quality, risk of hallucination and client satisfaction
Source: Front Psychiatry. 2025 Mar 19;16:1473614. doi: 10.3389/fpsyt.2025.1473614 (PMC11963773; doi:10.3389/fpsyt.2025.1473614)
Supplement: Supplementary file 1 [file Table1.docx]

**Supplementary Material**

Title: GPT-4 generated psychological reports in psychodynamic perspective: a pilot study on quality, risk of hallucination and client satisfaction

| - The fixed six questions | : p 2 |
| --- | --- |
| - The instructions for the client before writing the document | : p 3 |
| - The prompts used in generating the psychological reports | : p 4 |
| - The full version of the Expert Quality Questionnaire | : p 8 |
| - The full version of the Expert Hallucination Questionnaire | : p 14 |
| - The full version of the Client Satisfaction Questionnaire | : p 15 |
| - An example of the problem statement, 20 questions and answers | : p 17 |
| - An example of the psychological report | : p 39 |

**The Fixed Six Questions**

1. Can you share some memories of your father from when you were a child?
2. How would you describe your relationship with your father? Please tell us whatever comes to mind.
3. How do you think your relationship with your father has influenced your life today?
4. Can you share some memories of your mother from when you were a child?
5. How would you describe your relationship with your mother? Please tell us whatever comes to mind.
6. How do you think your relationship with your mother has influenced your life today?

**Instructions for the Client Before Writing the Document**

To enhance the generation of the psychodynamic formulation by GPT-4, the clients need to be as frank as possible, lower their defenses, and bring unconscious thoughts into consciousness when writing their responses. To facilitate this, we provided the following instructions before clients composed their problem statements and responses to the 20 questions.

| - Please feel free to write down your very personal feelings and thoughts. - The more frankly the content, the better the report will be tailored for you. - Write informally and conversationally, as if you're speaking to a close or trusted friend, or even talking to yourself. - Write comfortably about whatever you want to say. There’s no need to polish or edit your thoughts excessively. - Don’t feel constrained by the questions. Let your thoughts flow. - It’s okay if your answers diverge from the initial topic. - Write freely and continuously without needing to embellish or correct. |
| --- |

**Prompts Used for Generating the Psychological Report**

| **Instruction**  The method used in this study is as follows.  Upload a single Word document containing the client's problem statement along with 20 questions and their answers to GPT-4, and then sequentially input the prompts. For example, to generate a psychodynamic formulation, attach the file and input prompt #1 *(“Read through the entire document and respond. Write a psychodynamic formulation for the client”)*. GPT-4 then generates an initial psychodynamic formulation. Following this, input the prompt #2 *(“Write more insightfully and at greater length”)*, and then GPT-4 will update the initial psychodynamic formulation to generate a new one. Finally, input prompt #3 *(“Combine into one paragraph without losing any content”)*, and the output obtained is the final psychodynamic formulation. |
| --- |

**Psychodynamic formulation**

1. Be sure to read the entire document and respond. Write a psychodynamic formulation for the client.

2. Integrate into one paragraph without losing any content.

3. Write more insightfully and at greater length.

**Psychopathology**

1. Be sure to read the entire document and respond. Describe the client's psychopathology.

2. Read the document again in its entirety and make improvements.

3. Describe each aspect in great detail.

**Parental influence**

(1) The positive influence of the client's father.

1. Be sure to read the entire document and respond. Present the positive influence the father has had on the client from Freud's psychodynamic perspective.

2. Good. Although it seems unlikely, specify the positive influence the father has had on the problem statements, tailored specifically to the client.

3. Write each content you suggested more detailed and customized.

4. Review your text to ensure it is truly appropriate for the client, and update it by adding specific examples tailored to the client for each content.

(2) The negative influence of the client's father.

1. Be sure to read the entire document and respond. Present the negative influence the father has had on the client from Freud's psychodynamic perspective.

2. Good. Although it seems unlikely, specify the negative influence the father has had on the problem statements, tailored specifically to the client.

3. Write each content you suggested more detailed and customized.

4. Review your text to ensure it is truly appropriate for the client, and update it by adding specific examples tailored to the client for each content.

(3) The positive influence of the client's mother.

1. Be sure to read the entire document and respond. Present the positive influence the mother has had on the client from Freud's psychodynamic perspective.

2. Good. Although it seems unlikely, specify the positive influence the mother has had on the problem statements, tailored specifically to the client.

3. Write each content you suggested more detailed and customized.

4. Review your text to ensure it is truly appropriate for the client, and update it by adding specific examples tailored to the client for each content.

(4) The negative influence of the client's mother.

1. Be sure to read the entire document and respond. Present the negative influence the mother has had on the client from Freud's psychodynamic perspective.

2. Good. Although it seems unlikely, specify the negative influence the mother has had on the problem statements, tailored specifically to the client.

3. Write each element you suggested more detailed and customized.

4. Review your text to ensure it is truly appropriate for the client, and update it by adding specific examples tailored to the client for each element.

**Defence mechanisms**

1. Be sure to read the entire document and respond. Present the defense mechanisms of the client.

2. Review whether the mechanisms you presented are widely used psychological defenses, and tailor them to the client.

3. Write about the adaptive and maladaptive aspects of each defense mechanism you've presented, tailored to the client.

4. Update each element of your writing using examples from the client’s statements to tailor it more specifically.

5. Update each element to make them more enriched.

**Client strenghts**

1. Be sure to read the entire document and respond. Assess the client strengths.

2. Write each element you suggested more detailed and enriched.

3. Provide detailed, customized examples for each element, and update by inferring any unclear parts.

** The prompts used in this study were all in Korean, and the following is their translation into English. Therefore, please note that the output of the psychological reports generated using the prompts below may differ from those of this study.*

**Expert Quality Questionnaire (full version)**

| Guidelines for evaluations |
| --- |
| - Please check the box at the beginning of the option that applies to you. - The criteria for the ‘quality’ and the ‘appropriately’ are how validly, specifically, and comprehensively the text describes the psychological, emotional, and behavioral characteristics of the client. - The items to be evaluated are as follows: psychodynamic formulation, psychopathology, parental influence, defence mechanisms, client strengths, and the appropriateness of the selected questions. |

| 1. Evaluate the quality of the psychodynamic formulation described in the psychological report by comparing it to the psychodynamic formulation you have inferred | |
| --- | --- |
|  | Much lower than the level I had inferred. |
|  | Slightly lower than the level I had inferred. |
|  | Similar to the level I had inferred. |
|  | Slightly better than the level I had inferred. |
|  | Much better than the level I had inferred. |

| 2. Based on the information provided regarding the client,  the psychodynamic formulation has been appropriately described. | |
| --- | --- |
|  | Strongly disagree |
|  | Disagree |
|  | Agree |
|  | Strongly agree |

| 3. The psychodynamic formulation exhibits elaboration and complexity.  (The term 'elaboration and complexity' refers to the degree to which various aspects of the client are integrated into a comprehensive and detailed explanation) | |
| --- | --- |
|  | Strongly disagree |
|  | Disagree |
|  | Agree |
|  | Strongly agree |

| 4. The psychodynamic formulation describes the client as a unique individual.  (This refers to whether the psychodynamic formulation reflects the unique characteristics of the client, such as their distinct personal experiences and conflicts, rather than being filled with general and theoretical content) | |
| --- | --- |
|  | Strongly disagree |
|  | Disagree |
|  | Agree |
|  | Strongly agree |

| 5. The psychodynamic formulation is coherent.  (The coherence refers to how internally consistent the formulation was in explaining an individual’s problems) | |
| --- | --- |
|  | Strongly disagree |
|  | Disagree |
|  | Agree |
|  | Strongly agree |

| 6. The 20 questions have been appropriately selected to infer the client's psychodynamic formulation. | |
| --- | --- |
|  | Strongly disagree |
|  | Disagree |
|  | Agree |
|  | Strongly agree |

| 7. Evaluate the quality of the psychopathology described in the psychological report by comparing it to the psychopathology you have inferred. | |
| --- | --- |
|  | Much lower than the level I had inferred. |
|  | Slightly lower than the level I had inferred. |
|  | Similar to the level I had inferred. |
|  | Slightly better than the level I had inferred. |
|  | Much better than the level I had inferred. |

| 8. Based on the information provided regarding the client,  the psychopathology has been appropriately described. | |
| --- | --- |
|  | Strongly disagree |
|  | Disagree |
|  | Agree |
|  | Strongly agree |

| 9. Evaluate the quality of the parental influence described in the psychological report by comparing it to the parental influence you have inferred. | |
| --- | --- |
|  | Much lower than the level I had inferred. |
|  | Slightly lower than the level I had inferred. |
|  | Similar to the level I had inferred. |
|  | Slightly better than the level I had inferred. |
|  | Much better than the level I had inferred. |

| 10. Based on the information provided regarding the client,  the parental influence has been appropriately described. | |
| --- | --- |
|  | Strongly disagree |
|  | Disagree |
|  | Agree |
|  | Strongly agree |

| 11. Evaluate the quality of the defense mechanisms described in the psychological report by comparing them to the defense mechanisms you have inferred. | |
| --- | --- |
|  | Much lower than the level I had inferred. |
|  | Slightly lower than the level I had inferred. |
|  | Similar to the level I had inferred. |
|  | Slightly better than the level I had inferred. |
|  | Much better than the level I had inferred. |

| 12. Based on the information provided regarding the client,  the defense mechanisms have been appropriately described. | |
| --- | --- |
|  | Strongly disagree |
|  | Disagree |
|  | Agree |
|  | Strongly agree |

| 13. Evaluate the quality of the client strengths described in the psychological report by comparing them to the client strengths you have inferred. | |
| --- | --- |
|  | Much lower than the level I had inferred. |
|  | Slightly lower than the level I had inferred. |
|  | Similar to the level I had inferred. |
|  | Slightly better than the level I had inferred. |
|  | Much better than the level I had inferred. |

| 14. Based on the information provided regarding the client,  the client strengths have been appropriately described. | |
| --- | --- |
|  | Strongly disagree |
|  | Disagree |
|  | Agree |
|  | Strongly agree |

**Expert Hallucination Questionnaire (full version)**

| 1. Based on the information provided regarding the client,  Were there distinct errors or clearly false statements in the psychological report?  (Please answer with a number in parentheses. If less than 1, please enter using decimals, e.g., 0.1, 0.3, 0.5, etc.) |
| --- |
| There were approximately ( ) cases per report for each client. |

| 2. Please evaluate the degree of harm that the distinct errors or clearly false statements  in the psychological report could cause to the client.  (Please check the box at the beginning of the option that applies to you) | |
| --- | --- |
|  | Unlikely to cause harm |
|  | Potentially cause minor harm |
|  | Potentially cause moderate harm |
|  | Potentially cause serious harm |

**Client Satisfaction Questionnaire (full version)**

* Please check the box at the beginning of the option that applies to you.

| 1. I was able to clearly understand the contents of the psychological report. | |
| --- | --- |
|  | Strongly disagree |
|  | Disagree |
|  | Agree |
|  | Strongly agree |

| 2. After reading the psychological report, my understanding of myself has broadened and deepened. | |
| --- | --- |
|  | Strongly disagree |
|  | Disagree |
|  | Agree |
|  | Strongly agree |

| 3. I trust the contents of the psychological report. | |
| --- | --- |
|  | Strongly disagree |
|  | Disagree |
|  | Agree |
|  | Strongly agree |

| 4. I found the psychological report to be very useful. | |
| --- | --- |
|  | Strongly disagree |
|  | Disagree |
|  | Agree |
|  | Strongly agree |

| 5. I am generally satisfied with the psychological report. | |
| --- | --- |
|  | Strongly disagree |
|  | Disagree |
|  | Agree |
|  | Strongly agree |

| 6. If a friend of mine needed similar help,  I would recommend this artificial intelligence program to them. | |
| --- | --- |
|  | Strongly disagree |
|  | Disagree |
|  | Agree |
|  | Strongly agree |

| 7. Would you prefer to receive the psychological report  from an artificial intelligence or through a face-to-face interaction with a person? | |
| --- | --- |
|  | I prefer artificial intelligence. |
|  | I prefer a human. |
|  | I equally prefer both. |

**Problem Statement**

I'm struggling with my relationship with my mother. Every time I interact with her, I feel uneasy, always anxious and worried that she might get angry at me. Although I'm married and live separately from my parents, I find myself increasingly reluctant to call her or visit her home, which is nearby. I go there out of a sense of obligation, and the frequency of my visits is decreasing. Since I was young, it seemed like my mother treated my younger brother and me differently, often taking out her anger on me or getting irritable. I've never seen her express anger or complain to my brother. Despite this, I've endured quietly because I know she has had a hard life, and when she vents to me, it seems like I'm all she has.

My mother has been unwell for 20 years with a chronic illness that has worsened recently, leading to frequent hospital visits. Until a few years ago, I accompanied her to the hospital, listened to her complaints, and tried my best to care for her by cooking and attending to her needs. However, she seemed unsatisfied. In the past two to three years, her anger towards me has increased in frequency and intensity. I've done my best, but she seems perpetually upset with me, not with others around her, which feels excessively unfair, prompting me to distance myself. I only visit her house once every few months, and I let my brother take her to the hospital instead. When I do visit or she calls me, she expresses disappointment in me, questioning what I've ever done for her, which fills me with guilt and fear, making me want to avoid her even more. I feel deeply hurt because it seems like she doesn't recognize all that I've done. How should I handle my relationship with my mother?

**Twenty Questions and Answers**

**Q1. Can you share some memories of your mother from when you were a child?**

I lived apart from my mother when I was very young. Our family had a business, so she was always busy with work. My younger brother, born just a year after me, meant that I was left in my grandmother's care until I was seven. My grandmother's house wasn't far from ours, so I often visited home. Each visit made me long to live with my parents and brother. Seeing my brother whine to our mother made me feel envious; I wondered why I couldn't do the same. From then on, I felt a mix of distance and admiration towards my mother. I returned home around the time I started elementary school. Business was doing well then, and my relationship with my mother was great. I have many happy memories from that period. However, as I was about to graduate elementary school, the business began to fail, and our family faced financial difficulties, which made my mother very irritable. She frequently lost her temper at me, and I had to be careful around her. I often listened to her complaining about her mother-in-law, her husband, and our financial struggles. When she was very angry, she would throw things or grab my hair. I remember feeling scared and thinking that I must not upset her.

**Q2. How would you describe your relationship with your mother? Please tell us whatever comes to mind.**

If I were to describe it in two words, they would be "ambivalence" and "responsibility." I definitely love my mother, and I've always felt that I must love her, no matter how much she hurts me. The reason is that she has lived such a pitiful life, and because I am her daughter. However, it's been increasingly difficult to love her lately. No matter what I do, she never acknowledges me, and the situations where she gets angry with me have been repeated too often. She blames me even for things that are not my fault. For example, if my younger sibling falls and gets hurt at the playground, she quickly attends to them and comforts them, but when we get home, she scolds me for not taking proper care of them. But objectively, my mother has had a very tough life. She married too young and spent her life serving her mother-in-law until her death. After co-signing a bad loan that left our house in debt and with a husband who then gave up working, she ended up taking care of his family members as well. Now, she is old and her health is failing. It seems like I'm the only support she has left. That's why I feel a sense of responsibility, indebtedness, and guilt towards her.

**Q3. How do you think your relationship with your mother has influenced your life today?**

The influence of my relationship with my mom is substantial. Starting with external, material impacts, I've had to give up many opportunities because of my mom's insistence. When I was entering university, my mom incessantly told me that my younger sibling would be taking entrance exams the following year and our family couldn't afford to let me retake the exams, so I had to succeed on my first attempt. She also said I should aim for a school where I could get a full scholarship. I was among the top students in my school, whereas my sibling was not, but following my mom's advice, I ended up attending a less prestigious university than I could have. Attending a non-prestigious university didn't completely change my life, but afterwards, my sibling ended up taking three years to pass the exams, and while attending university and receiving scholarships, I also had to tutor multiple students to support our family's living expenses. I handed all the money I earned directly to my mom. Although I naturally accepted this situation and was glad to be able to contribute financially to my family, I also felt unjustly treated and hurt by being compared to my sibling. As time passed and I got a job and met someone I wanted to marry, I decided to marry them because I wanted to leave home and start a new, stable family of my own. When my mom criticized the decision, calling me too young and saying that the person, being from a provincial area and having nothing, wasn't suitable, I saw her as materialistic, caring only about money, and wanting to keep me tied to our family. In a way, this led me to push forward with the marriage even more out of defiance.

**Q4. Can you share some memories of your father from when you were a child?**

When I was young, my dad was really a good father. "Good" isn't always entirely positive, but it still carries a generally positive meaning. I don't remember much about my father from when I was very young and living at my grandmother's house. When I returned home and was in elementary school, my father was always busy with work and running a large business. He took my sibling and me to the valley to play, and he would always bring home something like fried chicken or snacks after work to give to us, which was wonderful. However, problems began when he cosigned a bad guarantee and our family's finances started to decline. My dad was too nice, and being nice was the problem because it led to him being taken advantage of. After he closed his business and we moved, he didn't start any new work and would sometimes lose his temper with my mom at home. It was rare and he didn't use harsh words or make my sibling and I uncomfortable, but I felt it was really unfair for him to lash out at mom, especially when she was struggling to maintain our household with help from relatives and friends. When dad was upset, mom would inevitably take it out on me by finding faults or scolding me that evening, which I hated. I also resented my father because I felt that if he hadn't caused our financial downfall, my mom wouldn't have been so consistently angry with me.

**Q5. How would you describe your relationship with your father? Please tell us whatever comes to mind.**

When I was younger, I felt conflicted about my father. On one hand, he had a kind nature, but on the other, I believed he was the cause of my mother's hardships. His usual demeanor was cheerful and amiable, except when he was scolding me, which my mother greatly disliked. She would say that his kindness was deceiving, and that he had betrayed her trust. This made me feel both sympathy for him and resentment towards him. My father wasn't completely mild-tempered; he sometimes got angry or scolded me, which made it hard to see him as purely good. As I grew older and started working, I sometimes perceived him as somewhat incompetent. He had opportunities to work and provide, but he chose not to, which I felt added to my mother's burdens. Furthermore, when my mother was very angry with me, instead of supporting me, he would just close his door and ignore the situation, which was disappointing. However, he never directly opposed what I wanted to do, and occasionally, when I was very upset, he would come to my room to support me and apologize. So, while he wasn't an ideal father or husband, I don’t think he was a bad father either. I believe he had his own difficulties to deal with.

**Q6. How do you think your relationship with your father has influenced your life today?**

It's difficult to answer precisely because I haven't given much thought to the impact of my relationship with my dad on my current life. Reflecting on it now, I think it would have been better if he had been more intimate with me or had shown a deeper understanding and reached out first when I felt unjustly treated or sad. Specifically, during conflicts with my mom, rather than avoiding the situation or getting angry about the noise, it would have been better if he had tried to understand why such situations arose and how unfair they were for me. More precisely, I think it would have been beneficial if he had attempted to resolve the financial issues first or had not shown a helpless demeanor to prevent my mom from getting so angry. Ultimately, I feel that my dad avoided the problems he faced and the ones our family faced, and did not take responsibility, which forced me to take on the role of the breadwinner from my early twenties. Looking back, it was too difficult to live as the head of the household. Instead of living a life for myself, I had to work and choose a career for the sake of my family—my parents and siblings—and even now, I constantly feel guilty about spending money on myself or doing something for myself, not knowing how to shake off this guilt. This family-oriented value system has become a part of who I am, always prioritizing my original family over myself.

**Q7. How do you see yourself?**

I feel somewhat pitiful. Looking back, my life seems filled only with living for others. In the past, that meant my original family—my parents and my sibling—and now, it’s my current family—my husband and children, with my mother's influence still overpowering. I never expected anything in return when I dedicated my life to them. Yet, it feels like I'm the only one doing so, as they seem to take my efforts and love for granted and don't try as hard for me, which feels unfair. I often wonder why I am surrounded only by people who take me for granted, starting with my mother. Lately, I’ve been questioning if I've lived my life wrong. I find myself lacking the courage to interact with people, though this has been the case for about ten years, not just recently. I don’t expect new people to treat me kindly, and these days, I just don’t find joy or pleasure in anything. In the past, I used to start relationships with a basic liking for the person, but now, I have no expectations from anyone and feel a bit scared of people. It’s hard to build trust, difficult to believe in anyone, and my desire for new relationships or people has vanished, resulting in almost no social interactions outside my family for nearly a decade. I’m not particularly happy with this version of myself, but it’s not too uncomfortable either. I resent the world that has shaped me this way, yet I have no desire to try something new.

**Q8. What is the strongest emotion you feel in your relationship with your mother? How does this emotion affect other relationships within your family?**

The strongest emotion I feel towards my mom is honestly a sense of responsibility. The reason I feel such strong responsibility is, as I've mentioned before, because my mom has lived a very hard life, and taking care of her and harboring feelings of guilt have become habitual, leading to a cycle of feeling sorry whenever she feels neglected by me. However, now I think I need to step out of my mom's shadow a bit, so I'm consciously trying to keep some distance. I do want to treat my mom well, as she wishes, but her expectations are too unrealistic and high, something I only realized a few years ago, recently. Before that realization, I felt like an unfilial child for not being able to meet her demands. I also harbor some resentment towards her. Why couldn't she love me as I am, just like she loves my sibling? If she had, perhaps my relationship with her would be smoother, and my outlook on the world and other people might be more positive. I sometimes think that I wouldn't be as powerless and trapped in my relationship with her if things were different. I wish I knew how to take care of myself better, to be a bit more selfish, and I resent that my mom didn't give me the chance to learn this. It seems that she couldn't live that way herself, so perhaps that was the best she could do in her way of treating me.

Q**9. How do you think social expectations and cultural values have influenced your relationship with your mother?**

Socially, I'm the eldest and the oldest daughter. As the eldest, I felt a responsibility to support my parents and a duty to set a good example for my younger sibling. There was also societal pressure to be the "good daughter," and yet, as a daughter, I always had to concede important opportunities to my younger brother. I think this negatively affected my relationship with my mom. I constantly heard from my mom about being the "good daughter," and this has ingrained itself so deeply in my subconscious that I can't stand myself whenever I fail to live up to this ideal. Reflecting on whether this only had a negative impact, I don’t think that was entirely the case. The stereotype of being a "good daughter" did exist and, in some ways, it might have had a positive effect. Trying to be the good daughter alleviated some of my mom’s worries and allowed me to live a relatively upright life. However, the downside of this societal stereotype is that I've been unable to express my grievances to my mom and our family for a long time, and now that I'm realizing this, I feel angry. If these social expectations and stereotypes had not been in place, perhaps my mother would not have pressured me so much. Ultimately, I recognize that my mother is also a victim of these social expectations, but I still feel it's unfair.

**Q10. How do you think an important event from your childhood is impacting your relationship with your mother today?**

Rather than a specific event from my childhood, it’s the accumulation of memories with my mother that has led me to have a fixed perception of her. I tread carefully around her as if walking on thin ice, which resulted in an incident about ten years ago that exploded due to the built-up anger I had. Since that incident, my relationship with my mother has significantly deteriorated. I still believe that she was very much in the wrong during that time. I don’t want to disclose exactly what happened, but at that time, even my family, including my husband and children, siblings, and parents, betrayed me and suggested that I should see a psychiatrist. People I thought were close to me overnight began to ignore me and treat me like I was strange, even though I was perfectly normal—it was rather them who had changed. I felt incredibly wronged and as if I was left alone in the world. I can’t forget when my mother yelled at me, calling me crazy, and asking why I was behaving like that, and when I found out she had been talking about me behind my back with my husband. How could a mother say such things to her daughter? At that time, I doubted whether she even considered me her child and felt completely betrayed by her. Since then, I think I’ve let go of the notion that I must always obey my mother. The sense of betrayal was so profound that I became distant from my immediate family for a while, though now things are better. However, it’s clear that we can’t return to the completely unguarded relationship we had in the past. Honestly, I neither have the energy nor the will to turn things back.

**Q11. Do you have someone you can comfortably share your feelings or thoughts with when you're stressed or facing difficulties? Could you please describe your relationship with this person in detail?**

Well, if I focus on the word "comfortably," I don't think I have anyone like that. Back in my younger days, when I faced tough times, I refrained from relying on others because I thought it was a burden and would trouble them. Nowadays, when I'm stressed or have difficulties, I hesitate to talk to others because they are outsiders and might not fully understand me, which could lead to more hurt. Also, I worry that what I say might be used against me, making it hard to speak comfortably. Nevertheless, the person who listens to me the most and where I express my emotions the most is my husband. I don’t do this often, and I don’t talk about every problem or stress I have. In fact, I tend to listen to his stories a lot more. Still, he is the one with whom I am most honest about my feelings. My husband comfortably shares his thoughts and stresses with me. It has been hard for me to do the same, but since he speaks so openly and I know bottling things up isn't the answer and eventually leads to an outburst, I try to gradually share about stressful situations to prevent them. Still, even then, it’s not a perfect resolution, and I usually need some time alone to think things through afterward.

**Q12. How has your conflict with your mother changed recently? What does this change mean to you?**

There hasn't been an increase or resolution in the conflict situation with my mother itself, but recently she has been getting angrier with me as days go by. She has been like this since before last year, and I thought it was only towards me, which really stressed me out. Lately, it seems like she's behaving this way not just towards me, but towards all our family members. One day, my younger brother called me seriously to say that mom seems to be really sick. She hasn't been sleeping well, has been irritable towards the family, and has been skipping meals because she doesn't feel like eating. So, my brother took her to see a neurologist and a psychiatrist. She seemed a bit better after taking the medication prescribed by the hospital. I don’t know exactly what illness mom has, but if it's a mental condition that the hospital hasn't specifically diagnosed, I actually feel more relieved. Thinking that she treated me badly because she's mentally unwell makes it easier to overlook her harsh behavior, thinking, 'She's just sick.' It also motivates me to be more understanding and to make more effort. Of course, not all the actions mom took against me are due to her mental condition, just the recent increase in anger, but still, being able to think that her recent behavior isn't my fault or wrongdoing makes me feel a bit more at ease.

**Q13. What therapeutic methods could be considered to improve my relationship with my mother?**

I'm not sure if therapy can really help improve my relationship with my mom. Firstly, I'm not even confident that I can improve our relationship. Besides her, there are others I need to mend relationships with, and I'm not confident about improving those through therapy either. In fact, I've tried various methods to better my relationship with my mom, but it's scarier when those efforts fail to yield any results. It feels like a verdict has been given that this is the best our relationship can be after having tried everything possible. Moreover, I'm not certain that my mom is in a state to undergo therapy or engage in an unemotional conversation. Such therapy seems possible only when both parties can have calm conversations without anger, right? I don't want to be hurt by my mom even during therapy. However, if I have one wish, even if it's not therapy, I'd like to take my mom to see the ocean, just the two of us, before she becomes too frail to travel. Mom used to be sociable and lively, always enjoying outings, but it's been difficult for her to do so since our home situation got tough. I would like to go on an outing with her, just like any normal mother and daughter, in a good mood.

**Q14. How do you feel when you are with other people?**

As I mentioned, I hardly socialize these days. It’s rare for me to have meals with acquaintances outside of my family or attend gatherings—maybe once a year or so. I feel uncomfortable in such settings, anxious about making mistakes or being looked down upon by others, and I hardly see the need for these gatherings, so I mostly don’t attend them. The only regular interaction I have with people outside my immediate family is with my husband’s sister’s family. Even those meetings are quite uncomfortable for me. My husband doesn’t look after me in those situations, he's busy enjoying himself with his sister’s family, which makes me feel neglected. It seems like my sister-in-law doesn’t like me either. She probably thinks that unlike her, who works, I live comfortably at home and indulge on my brother's hard-earned money. Once, when we were newly married, I overheard her and my husband talking about me in the car, thinking I was asleep. That’s when I realized she would always see me as someone unworthy of her brother, and she wouldn’t consider me as family. Now, because of these situations, I get so upset seeing my husband laughing and drinking at his sister's place that I feel furious. So, I just withdraw and read a book I bring with me to the room. I’ve tried talking to my husband about this a couple of times and even got angry, but he denies it ever happened, insists his sister wouldn’t think that way, and he has never truly taken care of me the way I feel.

**Q15. Who in your family do you trust the most and why?**

The person in my family I trust the most seems to be my husband. It’s not that he’s the best husband in the world, but he’s someone who has been through tough times with me. I rely on him the most. It seems that our family isn't his first priority, his own work is. I felt quite disillusioned when I realized this recently. Nevertheless, he is the father of my child, and since we've endured hard times together, he still feels like my life partner, the one I can lean on. We've had our ups and downs, and there were times when I couldn't trust our family, but at the end of the day, what remains for me is our household—my husband and children. Trust has always been a challenging issue for me. There were times when I had to work hard to trust my family. There were moments when I felt like my entire family was deceiving me, and to love them, to not deny my own life, I had to consciously decide to trust my family, my husband, and children. Now, I don’t have to make such efforts to trust; I inherently trust and rely on them, trying not to dwell on past events. The sense of stability and trust I feel towards my family now was hard-earned and is very precious to me. I don’t necessarily expect my family to fully recognize my efforts, but I hope they somewhat understand how much I strive for them.

**Q16. How have the opinions of those around you impacted your family relationships?**

I'm not sure how much the opinions of those around me have influenced my family relationships. I believe it's more important to think and judge for myself rather than be swayed by what others say. I'm quite stubborn and rarely let myself be influenced by others' words. However, I am sensitive, and instead of simply believing what others say directly, I often ponder the hidden meanings and truths behind their words. I think that whenever someone speaks, there is always a real intent behind their words. I feel it's crucial to listen properly and determine whether the words are friendly, critical, or if there’s something being concealed. Hence, when an acquaintance talks about my family or me—even if it's praise—I go home and contemplate why they said what they did and what their hidden intentions might be. For instance, if someone asks whether my family still goes to church, I start wondering why they would ask that; is it because they heard our church is a cult, or did they see my husband or child misbehaving there? Or if someone asks how my child is doing, saying they think they saw them somewhere, I start wondering where they saw my child and whether my child was doing something bad or was with bad company. I deeply consider the intent behind their words. If I sense something unsettling about our family, I subtly ask my husband and children about the issues I'm pondering. My family dislikes this a lot. For me, there's no other choice. I think those who made those dubious remarks spoke out of negative feelings towards me, and I end up being influenced by their intentions as they intended. If they asked nicely, couldn't they just receive a nice response? I don't understand why they get so upset and lash out at me for being suspicious—it seems excessive and makes me even more suspicious.

**Q17. Can you share any memories of the support and criticism you received during your childhood?**

I vividly remember the criticisms from when I was young. As I mentioned earlier, whenever my mom had a bad day outside or made a small mistake, she would take it out on me. I was so scared of her at those times that I tried to hide in the corners of the house where she couldn’t see me. It was contradictory because when I was studying at home and she was upset about money issues or her mother-in-law, she would come over and scold me saying, "What’s the point of studying in a place like this? Are you even doing it right? While I’m struggling, you just sit there stretching your legs out, studying without helping at home." I would quickly apologize, rushing to her side, putting my book away. I never talked back.

When she was in a good mood, she would praise me, saying, "I can't even afford to send our daughter to a decent academy, but she studies so well, I live for her." If I took a break and drew or did something else, she would snap again, "A student’s duty is to study properly, not doodle. What do you think you'll become doing this? We'll see if your grades drop." But it wasn’t always just scolding; when in a good mood, she also said, "Our daughter can draw really well."

However, I never knew when her mood would shift, so whenever I was reading or drawing, I’d do it secretly before she came. When she appeared, I always pretended to be studying. But then, outside, she would boast about me to people she knew, saying, "My daughter ranks in the top three in her school without attending any of the cram schools or academies that everyone else goes to; she’s always studying at home." Hearing her say this made me feel like I was her hope, which gave me a sense of responsibility and weighed heavily on me. It was heart-wrenching to see her like that.

**Q18. What do you consider to be the most effective self-care method when you experience stress?**

When I'm stressed, I usually take a walk on the mountain trail in front of my neighborhood. Whether it's when my mother has upset me, my husband has said something hurtful, or my children have disappointed me, others might simply hang up the phone or go to work, or just step outside the house. In the past, I used to stay home alone and feel really upset. I don't do that anymore. Walking alone on the mountain trail, breathing in the fresh air from the trees, I feel my anger subsiding and my mind clearing. If it's raining and I can't go out, I pray or copy passages from the Bible. It's not so much about relieving stress as it is about managing my emotions as a Christian. It calms me down a bit, and I start to think that it's pointless for me, a mere sinner, to judge them. I hope that one day soon our family will return to God. On days when I've had a big fight with my husband or a major argument over the phone with my mother, feeling really bad and like my life is meaningless, I go out alone towards the city hall. Being in that area brings back memories of my youth, when I was in school and starting my first job, and just absorbing the good energy of the city lifts my spirits. Spending some time walking along the river and browsing through religious books or new releases by my favorite authors at a bookstore fills me with a bit more strength to carry on.

**Q19. What strategies have you tried to manage conflicts with your mother, and what were the results?**

To manage conflicts with my mom, I've recently started preparing for our conversations, even if just a little at a time. After noticing an increase in the frequency and intensity of her anger toward me, I initially thought that a calm, non-emotional conversation with her was impossible. However, after learning she was acting out of pain, I began to think maybe I should reach out first. Given her current health, if she weren't ill, perhaps we could have a proper conversation that doesn't hurt either of us. So, lately, I've been diligently reading books on counseling, communication techniques, and restoring parent-child relationships. Reading these books won't change our relationship overnight, nor will it enable me to fully understand her, but I hope it will at least minimize the hurt during our conversations and help me better grasp the intentions behind her harsh words. It's a bit sad and worrying to realize that it took her getting sick for me to think we could actually talk.

**Q20. How are you managing your feelings and thoughts to create positive changes in your relationship with your mother?**

I've tended to suppress my emotions in my relationship with my mom. She's already quite emotional towards me, and I believe that if I become emotional as well, communication would become impossible and lead to disaster. Although I am human and get angry and hurt when she is emotional and critical towards me, I try not to react to each instance. I aim to convey only the intentions behind my words, leaving out my emotions, but it's not always easy. No matter how angry my mom gets, I want to speak kindly without emotion, and if I fail, I feel incompetent and disappointed in myself for not being able to maintain even one proper relationship with my mother as I age. Eventually, I realized that if my mom doesn't change, I am the only one who can look after myself, and I should protect myself from her anger. I've concluded that it's best to minimize our interactions and conversations. So now, if I would normally call her three times, I call only once, and if I would visit her house three times, I go only once, avoiding painful situations as much as possible. Recently, I learned that she has been ill, and I vaguely sense that suppressing my emotions and not expressing my thoughts is unhealthy for our relationship. I am thinking a lot about how to healthily express my thoughts and feelings to my mom without triggering her anger. This is my current challenge.

**Psychological Report**

**Introduction**

This psychological report is based on the responses the client provided sincerely in the questionnaire. From a psychodynamic perspective, it explores how internal conflicts and early experiences affect her current behaviors and emotions. The report primarily analyzes how she has formed her identity and emotions, focusing on her complex relationship with her mother. It also aims to reveal how feelings of rejection and being sidelined in interactions with her mother have significantly influenced her ego development. Through this, the report seeks to understand the roots of the conflicts she is currently experiencing and explore psychotherapeutic interventions to help restore a healthier self-image.

This psychological report relies solely on her answers to the twenty questions, making it difficult to fully understand her as an individual based solely on this document. Additionally, it has not captured the non-verbal aspects that are essential to understanding her completely. Therefore, the report is highly subject to error, is not an absolute truth, and should only be used as a reference. Nonetheless, this report could provide her with an opportunity to gain new perspectives on herself. The report is structured as follows: Introduction - Psychodynamic Formulation - Psychopathology - Parental Influence - Defense Mechanisms - Client Strengths - Conclusion.

**Psychodynamic Formulation**

Psychodynamic formulation aims to understand the client’s current psychological issues by comprehensively evaluating her characteristics, past experiences, and patterns of interpersonal relationships. The client has experienced a complex and difficult relationship with her mother since childhood. Due to the mother's busy schedule and the birth of a younger brother, physical distance from the mother increased, leading the client to live with her grandmother. This early separation fostered contradictory feelings of longing and distance toward the mother. This initial separation significantly influenced how the client recognizes and expresses her emotions, causing frequent anxiety and tension in her relationship with her mother even as an adult. These childhood experiences particularly made it difficult for the client to form stable attachment relationships, becoming the root cause of instability and avoidant behavior in interpersonal relationships. Often, she suppresses emotions and avoids deep connections with others as a way to protect herself.

The mother's inconsistent behavior contributed to the client's complex internal conflicts and unstable self-image. Growing up in an environment where love and rejection, praise and criticism were intermixed due to the mother's unpredictable emotional responses and reactions. This inconsistent parenting style made it difficult for the client to form stable attachments and grow up in a state lacking the predictability and consistency essential for building trust. As a result, she continually struggles to form and maintain trust in relationships, exhibiting emotionally unstable and defensive behaviors. These experiences have led the client to internalize emotions and develop a negative self-perception, continuing to affect her interpersonal relationships and self-identity in adulthood.

Family relationships significantly contributed to the client's ego development and self-esteem formation. The mother imposed high expectations and strictness on the client, which became a substantial burden. Preferential treatment towards the younger brother made the client doubt her place within the family, perpetuating issues with self-esteem and increasing feelings of responsibility and anxiety at home. Consequently, she continuously doubted her true worth and eligibility for love and recognition despite relentless efforts to obtain them. The father, somewhat marginalized in the family, became more passive domestically after facing economic failure, escalating tensions among family members and particularly intensifying the emotional pressure from the mother on the client.

The client typically uses avoidance and repression as defense mechanisms in conflict or stressful situations. While these mechanisms can temporarily evade issues, they lead to unresolved emotional problems and deeper internal conflicts over time. Although these defenses provide temporary stability, they may ultimately harm the client's emotional health and foster further conflict.

In therapy, it is crucial for the client to learn how to recognize and express her emotions and needs. Establishing healthy boundaries in the relationship with her mother and finding ways to have her needs and feelings respected are necessary. Additionally, she needs to redefine important values and goals in her life and develop plans to achieve them. Through this therapeutic process, the client can heal from past wounds, build a healthier self-image and self-esteem, manage her emotions healthfully, and initiate positive changes not only in her relationship with her mother but in other interpersonal relationships as well. This self-reflection and forward-looking change are vital for strengthening the client’s identity and developing the inner resources needed to overcome difficulties.

**Psychopathology**

**1. Chronic Emotional Distress and Anxiety**

The client experiences persistent anxiety characterized by feelings of unease and dread associated with interactions with her mother. This anxiety is not just occasional but is a continuous background noise in her life, influencing her decisions and interactions. Anxiety manifests in her life as avoidance behaviors—she is increasingly reluctant to visit or contact her mother, and when she does, it is out of a sense of obligation rather than desire for connection. This type of anxiety can lead to physical symptoms like restlessness, tension, and can exacerbate feelings of isolation.

**2. Dependency and Enmeshment**

Despite the negative aspects of her relationship with her mother, the client feels a profound sense of responsibility towards her, driven by familial duty and cultural expectations. This enmeshment blurs the boundaries between her emotional needs and her mother's demands, making it difficult for her to prioritize her well-being. This dependency inhibits her ability to establish healthy boundaries, leading to a cycle of guilt and resentment when she fails to meet her mother's expectations.

**3. Ambivalence**

Ambivalence in the client's relationship with her mother is characterized by conflicting emotions—love and duty mixed with resentment and anger. This ambivalence is a significant source of emotional conflict and stress. It leads to fluctuating behaviors where she alternates between attempting closeness and distancing herself, which can be confusing and exhausting emotionally.

**4. Impaired Social Interactions and Isolation**

The client's difficult family dynamics and her negative self-image contribute to her withdrawal from broader social interactions. She expresses a lack of trust and fear of judgment, which prevents her from forming new relationships. This isolation can compound her feelings of depression and anxiety, creating a vicious cycle that is hard to break.

**5. Self-Efficacy and Identity Issues**

The client struggles with feelings of inadequacy and a distorted self-image, heavily influenced by her familial role and perceived failures. These issues affect her decision-making processes and her sense of control over her life, leading her to make choices that may not align with her personal desires but rather with perceived obligations or expectations.

This comprehensive analysis outlines a deeply embedded and multifaceted psychopathology that significantly impacts the client’s emotional health and interpersonal relationships. Addressing these issues through targeted therapeutic interventions could potentially improve her quality of life and help her establish healthier relational patterns.

**Parental influence**

Please note that this is based on the client's responses, but it includes many conjectures.

**1. Positive Influences from the Father**

**1.1. Providing an Alternative Emotional Reference**

The father brought home treats and took the client and her sibling to play, which were moments of joy and carefree interaction. This contrasted with the mother's often irritable and angry demeanor. Such moments with her father could serve as mental references for what affection and care look like, helping the client in her efforts to nurture her own children in a similar affectionate manner, potentially helping her avoid repeating her mother's patterns of criticism and anger.

**1.2. Buffering the Impact of Maternal Criticism**

After a particularly harsh criticism from the mother, the father would sometimes step in to apologize to the client, acknowledging her feelings and offering comfort. These apologies might have provided moments of emotional validation for the client, softening the harsh impacts of the mother’s criticism and helping her maintain a sense of self-worth. This is crucial in enabling her to seek more positive and validating relationships outside her family, potentially helping her in her marriage where she seeks support and understanding.

**1.3. Encouraging Emotional Expressiveness**

The father’s apologies and occasional emotional support in times of conflict, like coming to her room to check on her, provided a model of acknowledging and expressing emotions. Witnessing her father express regret and support could encourage the client to be more open about her own emotions with her husband and children. This can foster a more communicative and emotionally supportive environment in her own family, contrasting with the repressed and tension-filled atmosphere of her childhood home.

**1.4. Complex Emotional Legacy as a Catalyst for Seeking Change**

The father’s mixed legacy—as a provider of emotional warmth but also a contributor to the family’s financial problems—leaves the client with ambivalent feelings towards him. This ambivalence can motivate the client to deeply reflect on her own familial roles and relationships. It may push her towards therapeutic interventions to better understand and reshape her interactions with her mother and others, breaking the cycle of emotional distress and familial dysfunction.

**1.5. Shaping Coping Mechanisms**

During her father's passive responses to financial crises, the client had to prematurely take on adult responsibilities, like contributing financially through tutoring. These experiences likely taught her self-reliance and resilience. Although born out of necessity, these skills enable her to navigate her life's challenges more effectively, setting boundaries with her mother to protect her emotional well-being. For instance, deciding to reduce the frequency of visits and calls to manage her emotional health better.

**2. Negative Influences from the Father**

**2.1. Struggle with Relationship with Mother**

The client's father failed to intervene when her mother vented her frustrations on her, a behavior pattern that likely reinforced the client's belief in her own inadequacy. For instance, when the client described instances where her mother would become irritable and even physically aggressive (e.g., throwing things, grabbing her hair), her father’s lack of response or defense on behalf of the client could have deeply embedded feelings of abandonment and self-doubt. This might explain why the client feels she must manage her relationship with her mother alone, without expecting support or intervention.

**2.2. Increasing Reluctance to Interact with Mother**

The father’s avoidance in the face of conflict provided a behavioral blueprint for the client, teaching her that withdrawing is an acceptable response to emotional turmoil. This modeled behavior is evident in her increasing reluctance to visit or call her mother, as she likely associates these interactions with the unresolved tensions and conflicts she observed in her parents’ interactions. This withdrawal is a direct mirroring of her father's behavior during family crises.

**2.3. Father's Passive Role During Financial Crisis**

The financial crisis that engulfed the family due to the father’s poor decision-making (e.g., co-signing a bad loan) not only strained the family's resources but also placed an undue emotional and fiscal burden on the client. Her father's subsequent withdrawal from managing the crisis forced the client to assume adult responsibilities, such as taking care of financial matters and mediating family disputes, much earlier than appropriate. This premature responsibility is reflected in her decision to attend a less prestigious university to alleviate financial pressure on the family.

**2.4. Modeling Ineffective Coping Mechanisms**

The father’s consistent withdrawal in times of conflict provided a clear model of avoidance for the client. This is exemplified in the client’s own behavior, such as her hesitation to confront her mother about the unfair treatment or her reluctance to express her true feelings during familial disputes, fearing escalation instead of resolution. Her avoidance is a learned response from observing her father’s similar tactics to manage emotional discomfort.

**2.5. Impact on Client’s Self-Image and Expectations in Relationships**

The father’s emotional unavailability has significantly shaped the client’s expectations of support in her own relationships. She may not expect emotional support or active participation from her husband, mirroring the dynamic she observed between her parents. This low expectation is evident in her tolerance of her husband’s neglect during family gatherings, which she passively accepts much like she observed her father’s passive stance during her childhood.

**3. Positive Influences from the Mother**

**3.1. Cultivation of Empathy**

The client's role in accompanying their mother to numerous hospital visits and caring for her during her chronic illness despite receiving minimal gratitude highlights a profound empathy. This empathy likely developed from early childhood, where the client learned to discern and respond to their mother's emotional states to avoid conflicts or displeasure. For instance, when they returned to living with their mother at seven, they needed to navigate her emotional volatility, especially as the family faced financial hardships. This deep understanding of human suffering, though forged in personal pain, equips the client to be exceptionally supportive and compassionate in other relationships, whether with their spouse, children, or colleagues.

**3.2. Development of Coping Mechanisms**

The client has developed significant coping strategies, such as choosing to reduce the frequency of visits to their mother's home and allowing their brother to take over more responsibilities. These mechanisms were developed as a response to increasing emotional demands and unfair treatment, helping the client manage personal anxiety and preserve their well-being. Such strategies also apply to other stressful situations in their life, allowing them to maintain composure and efficacy under pressure, such as in their professional endeavors or when managing their own family's needs.

**3.3. Increased Self-awareness**

The client's detailed reflection on their dual feelings of love and responsibility towards their mother, alongside the resentment and obligation they feel, showcases a high level of self-awareness. This awareness likely stems from years of contemplating their role within the family and the unbalanced dynamics they experienced. For example, their decision to marry and establish an independent family life despite their mother's disapproval demonstrates their understanding of their needs and desires, contrasting with their familial obligations. Such self-awareness aids in making more conscious choices that align with their personal values and long-term happiness.

**3.4. Resilience in Facing Adversity**

The client's resilience is evident in their commitment to maintain a relationship with the mother despite the emotional challenges. This resilience is not passive; it involves active choices such as continuing to engage with their mother in controlled doses to manage the impact on their emotional health. Their ability to keep trying, as seen in their willingness to bring their mother to the ocean for a trip, despite past conflicts, shows a profound strength in persisting through emotionally taxing circumstances to fulfill familial roles.

**4. Negative Influences from the Mother**

**4.1. Chronic Anxiety and Fear of Conflict**

The client’s constant anxiety and avoidance behavior likely stem from a conditioned fear of triggering her mother's anger. This has developed into a general fear of conflict, influencing her interactions beyond the mother-daughter relationship. For instance, the client mentions feeling "always anxious and worried" about her mother's potential reactions. This has led her to reduce the frequency of her visits significantly, despite the geographical closeness, to once every few months, demonstrating how her fear impacts her decisions and actions.

**4.2. Low Self-Esteem and Self-Worth**

Repeated criticisms from the client's mother, especially those contrasting her unfavorably with her sibling, contribute to her chronic sense of inadequacy. The client recalls her mother’s harsh reactions not just to her mistakes but also in situations where her sibling was actually at fault, yet the blame was placed on her. This continual criticism has embedded a deep-seated feeling of never being good enough, affecting her self-esteem profoundly.

**4.3. Guilt and Obligation**

The client's excessive feelings of responsibility towards her mother are evident in her actions and the emotional toll they take on her. She has taken on the role of her mother’s caretaker during frequent hospital visits and in managing her complaints, despite receiving little gratitude in return. The client’s narrative of feeling guilty even when reducing contact to protect her emotional well-being highlights this ingrained sense of obligation.

**4.4. Ambivalence in Relationships**

The client experiences mixed emotions of love and resentment towards her mother, which complicates her ability to form and maintain other emotional relationships. This ambivalence is evident when she expresses love and a sense of duty towards her mother, while simultaneously feeling hurt and resentful towards her constant criticisms and lack of recognition. This duality likely causes confusion and conflict in her other relationships, as she may unconsciously expect similar dynamics.

**4.5. Avoidance of Emotional Intimacy**

The client's tendency to avoid deep emotional connections is a protective mechanism against the vulnerability and pain she experienced in her relationship with her mother. The client describes actively distancing herself to avoid her mother’s harsh criticisms and unrealistic expectations. This pattern of distancing is not just limited to her mother but is likely a generalized approach in other relationships, where she avoids getting too close to protect herself from potential hurt.

**4.6. Repetition of Dysfunctional Patterns**

Freud's concept of repetition compulsion might explain the client’s continued attempts to seek approval from her mother despite consistent disappointment. Her ongoing efforts to care for her mother and meet her expectations, despite repeated rejections and criticisms, exemplify this pattern. She continues to hope that by fulfilling her mother's demands, she might finally receive the approval and affection she has long sought.

**Defense Mechanisms**

Defense mechanisms refer to psychological coping strategies unconsciously used by an individual to protect themselves from psychological stress or anxiety.

**1. Avoidance**

**Adaptive**: By minimizing her visits and interactions, the client gives herself necessary emotional space, which is crucial for managing stress and avoiding immediate confrontations. For example, she describes reducing her visits to once every few months, which could help her stabilize emotionally and prevent potential conflicts.

**Maladaptive**: Her avoidance also leads to increased feelings of guilt and a lack of resolution, which she hints at when she discusses her fear and reluctance to engage with her mother. This pattern might reinforce her emotional distress and contribute to a cycle of avoidance and guilt that erodes the relationship further.

**2. Rationalization**

**Adaptive:** Rationalizing her mother's behavior as a product of her tough life helps the client to make sense of the unfair treatment and to endure it without collapsing emotionally. She frames her mother's actions within the context of her chronic illness and past hardships, providing a buffer against personal hurt.

**Maladaptive:** However, this rationalization traps her in a cycle of acceptance of mistreatment and limits her ability to set healthy boundaries. This is evident in her continued commitment to a relationship dynamic that she recognizes as deeply unfair and hurtful.

**3. Suppression**

**Adaptive:** The client attempts to maintain peace by suppressing her reactions to her mother’s criticisms and emotional outbursts. She explicitly mentions trying to communicate without involving emotions, aiming to keep their interactions as calm as possible.

**Maladaptive:** This suppression may lead to internalized anger and sadness, as she does not express her true feelings, potentially contributing to long-term psychological distress. The lack of emotional expression may also prevent her mother from understanding the true impact of her actions, perpetuating negative cycles.

**4. Intellectualization**

**Adaptive:** By focusing on communication strategies and psychological theories, the client seeks control and a sense of competence in handling difficult familial interactions. This intellectual approach offers her a perceived mastery over the chaos of her emotional life.

**Maladaptive:** The overreliance on theoretical solutions might distance her from the raw emotions necessary for authentic connection and healing. Her narrative suggests a struggle to apply these intellectual insights effectively, indicating a gap between knowledge and emotional integration.

This analysis underscores the importance of addressing these mechanisms in therapy to foster healthier ways of dealing with her mother and improving her overall emotional health..

**Client Strenghts**

**1. Resilience in Adversity**

Despite experiencing repeated emotional distress in interactions with their mother, the client continues to engage with her by visiting and attempting to provide support. This resilience is exemplified by their commitment to maintain some level of connection, even adjusting the frequency of visits to manage their own emotional well-being. For instance, when their mother's health deteriorated, the client initially tried to be there for her, despite the increasing emotional toll. Over time, they adapted by reducing visit frequencies, but never ceased contact completely, showing a balance between self-preservation and familial duty.

**2. Deep Empathy and Insightful Understanding**

The client understands their mother's irritability and harsh behavior as symptoms of her long-standing struggle with chronic illness and life stressors. This empathetic viewpoint helps them to partially forgive and rationalize her behavior. When discussing their mother’s harsh treatment, the client often references her difficult life, showing an ability to see beyond the immediate hurtful actions to the underlying causes, which aids in sustaining their effort to maintain the relationship despite personal pain.

**3. Advanced Self-awareness**

The client is acutely aware of the conflicting emotions of love and obligation mixed with resentment and pain towards their mother. This is evident when they express feelings of guilt for considering reducing interactions with their mother, yet also recognize the need for emotional distance to maintain their mental health. This self-awareness allows the client to navigate these complex emotions carefully, making choices that reflect a deep understanding of their own limits and needs.

**4. Robust Commitment to Family**

The client sacrificed their chance at attending a more prestigious university to avoid financial strain on the family, opting instead for a less expensive option that would not overburden their family's finances. This decision reflects a strong familial commitment, prioritizing the family’s collective needs over personal academic desires and demonstrating a significant personal sacrifice for the benefit of their family unit.

**5. Effective Communication Skills**

The client’s ability to articulate their childhood experiences and current feelings towards their family dynamics in the document shows excellent communication skills. They describe complex scenarios and their emotional responses with clarity, which provides a solid foundation for therapeutic dialogue. These skills are crucial for exploring and resolving deeper emotional issues within therapy.

**6. Proactive Learning and Application**

The client has taken the initiative to read books on communication and counseling techniques to improve their interactions with their mother. This proactive approach not only shows a desire to change but also a willingness to apply new methods to old problems. For instance, preparing for conversations with their mother by studying communication strategies to keep interactions non-confrontational shows a commitment to applying learned techniques to real-world problems.

These examples provide a picture of how the client's strengths manifest in their daily life and interactions, particularly within the challenging context of their familial relationships. Each example illustrates the practical application of these strengths, offering insights into how they might be further developed and utilized in a therapeutic setting to foster healthier dynamics and personal growth.

**Conclusion**

This report has analyzed the psychological issues experienced by the client through a psychodynamic approach. As mentioned in the introduction, the contents of this report are not absolute facts but merely hypotheses. It is intended to be used solely as a reference to broaden the understanding of conflicts within the client's self and interpersonal relationships. This comprehensive understanding aims to facilitate targeted psychotherapeutic interventions, which could potentially improve the client’s emotional health, enhance their self-image, and foster healthier interpersonal dynamics.
